# Draft genome sequences of *Bacillus velezensis* and *Bacillus subtilis* strains with plant growth-promoting and drought resilience potential

**DOI:** 10.1128/mra.00516-25

**Published:** 2025-07-18

**Authors:** Lourenço Vitor Silva Ferreira, Ubiraci Gomes de Paula Lana, Christiane Abreu de Oliveira-Paiva, Eliane Aparecida Gomes, Sylvia Morais de Sousa Tinoco

**Affiliations:** 1 Federal University of São João del-Rei 74383, São João del Rei, Minas Gerais, Brazil; 2 Embrapa Maize and Sorghum 592341 , Sete Lagoas, Minas Gerais, Brazil; DOE Joint Genome Institute, Berkeley, California, USA

**Keywords:** drought, plant growth-promoting bacteria, Brazilian Caatinga, soil microbiology

## Abstract

*Bacillus* spp. are bacteria recognized for enhancing plant growth. This study presents the genomic characterization of *Bacillus velezensis* (strains 6E9 and 5D5) and *Bacillus subtilis* (strains 2E7, 1A11, and 1H10), isolated from soils of the Brazilian Caatinga biome. Genomic insights reveal their potential for bioproducts that enhance crop drought resilience.

## ANNOUNCEMENT

An economically beneficial and environmentally sustainable alternative to mitigate the effects of drought on different crops is the use of microbial inoculants formulated with plant growth-promoting bacteria (PGPB) (1). The use of PGPB in the formulation of biofertilizers can increase nutrient availability in the soil and protect plants from damage under drought stress conditions, providing a considerable improvement in water use efficiency (2, 3). Sequencing the new *Bacillus* strain is essential to explore its genetic potential for drought tolerance and to develop effective biofertilizers tailored to diverse agricultural needs.

Bacterial strains were isolated from soil samples collected in the Caatinga biome of Ceará, Brazil. For each sample, 1.0 g of soil was transferred to a 15 mL conical tube containing 5 mL of 0.85% (wt/vol) NaCl. After 15 h of constant shaking, 1.0 mL of the suspension was heated at 65°C, cooled on ice for 15 minutes, plated on tryptic soy agar, and incubated at 40°C for 24 h. Five bacterial strains, designated 6E9, 5D5, 1H10, 2E7, and 1A11, were isolated from Canindé, Tauá, Quixeramobim, Pedra Branca, and Ocara, respectively.

These strains were capable of growing under low water activity conditions (520 and/or 780 g/L sorbitol), producing exopolysaccharides in vitro, and synthesizing siderophores on chrome azurol S medium at neutral pH. Based on these traits, they were selected for whole-genome sequencing (WGS).

All strains were cryopreserved in a glycerol-based medium at −80°C and deposited in the Embrapa Collection of Multifunctional and Phytopathogenic Microorganisms for Maize and Sorghum (CMMF) under the following accession numbers: BRM051761 (CMPC2415) – 6E9, BRM051757 (CMPC2411) – 5D5, BRM051747 (CMPC2401) – 2E7, BRM051734 (CMPC2388) – 1A11, and BRM051737 (CMPC2391) – 1H10. 

Genomic DNA was extracted from pure bacterial cultures grown in Luria-Bertani liquid medium at 28°C for 24 h under agitation (150 rpm). Following incubation, cultures were centrifuged at 14,000 × *g*, and the supernatant was discarded. DNA from strains 5D5, 1H10, 6E9, 1A11, and 2E7 was extracted using the Wizard Genomic DNA Purification Kit (Promega, USA) and quantified with a Qubit 2.0 fluorometer (Life Technologies, USA). WGS libraries were prepared by using the Agencourt AMPure XP-Medium kit (Beckman Coulter, USA) to an average size of 200–400 bp. Sequencing was carried out on a BGISEQ-500 platform at the Beijing Genomics Institute (BGI), Shenzhen, China, using the protocol described in Huang et al. (4), and a 150 bp paired-end strategy. Base calling was performed using BGISEQ-500’s internal software. Raw reads were quality-filtered using Trimmomatic v0.38 (5) to remove adapters and low-quality bases (Phred score < 20). High-quality reads were assembled *de novo* with SPAdes v3.12.0 (6). Assembly quality was evaluated using QUAST v5.0.2 (7), and genome completeness was assessed with BUSCO v5.3.1 (Benchmarking Universal Single-Copy Orthologs) (8). All software was run with default settings.

Key genomic features of strains 6E9, 5D5, 1H10, 2E7, and 1A11 are summarized in Table 1. The genome sequences were submitted to National Center for Biotechnology Information (NCBI) and annotated using the Prokaryotic Genome Annotation Pipeline v6.6.1 (9). Annotation revealed 3,821, 3,739, 4,000, 4,073, and 4,332 coding sequences; 10, 11, 13, 11, and 18 rRNAs; and 84, 84, 87, 86, and 91 tRNAs in strains 6E9, 5D5, 1H10, 2E7, and 1A11, respectively. Additionally, one transfer messenger RNA (tmRNA) was identified in each genome.

**TABLE 1:** Genomic features of *Bacillus velezensis* (6E9 and 5D5) and *Bacillus subtilis* (1H10, 2E7, and 1A11)

Characteristic	*B. velezensis*		*B. subtilis*		
	6E9	5D5	1 H10	2E7	1 A11
Fast average nucleotide identity (ANI) placement (%)	98.10	98.07	98.35	98.51	98.38
Fast ANI reference (NCBI)	JS25R	JS25R	ATCC 11774	ATCC 11774	ATCC 11774
Library size	13,606,800	13,623,596	13,596,210	13,576,868	13,544,004
Number of contigs	23	19	19	16	22
Total sequence length (bp)	3,988,266	3,894,451	4,045,220	4,090,212	4,285,006
Total ungapped length (bp)	3,988,172	3,894,355	4,045,220	4,090,115	4,284,823
N50 (kb)	475,082	703,010	2,101,641	2,082,097	1,144,601
G+C content (%)	46.7	46.54	43.71	43.68	43.59
Genes	3,916	3,835	4,101	4,171	4,442
Protein-coding	3,821	3,739	4,000	4,073	4332
Non-coding (RNA)	95	96	101	98	110
Genome completeness (%)	99.81%	99.26%	99.81%	99.81%	99.81%
Genome contamination (%)	0.73%	0.07%	0.12%	0.12%	0.83%
Coverage (×)	489	499	480	476	453
SRA identifiers	SRS24780036	SRS24780037	SRS24780038	SRS24780039	SRS24780040
Genome assembly (NCBI)	SRX28458681	SRX28458682	SRX28458683	SRX28458684	SRX28458685
BioSample ID	SAMN48054946	SAMN48054947	SAMN48054948	SAMN48054949	SAMN48054950
GenBank acession number	JBNHMZ000000000.1	JBNHMY000000000.1	JBNHMV000000000.1	JBNHMX000000000.1	JBNHMW000000000.1
Strain	BRM 051761	BRM 051757	BRM 051737	BRM 051747	BRM 051734
Isolate name alias	CMPC 2415	CMPC 2411	CMPC 2391	CMPC 2401	CMPC 2388
Country/state /municipality	BRA/Ceará/Canindé	BRA/Ceará/Tauá	BRA/Ceará/Quixeramobim	BRA/Ceará/Pedra Branca	BRA/Ceará/Ocara
Geographic location	04º 21' 32" S, 39º 18' 42" W	06º 00' 11" S, 40º 17' 34" W	05º 11' 56" S, 39º 17' 34" W	04º 49' 56" S, 40º 19' 14" W	04º 18' 01" S, 38º 29' 52" W
Biome	Caatinga	Caatinga	Caatinga	Caatinga	Caatinga
Collection date	18 October 2013	17 October 2013	15 October 2013	16 October 2013	16 October 2013

For taxonomic classification based on genomic data, the Type (Strain) Genome Server (https://tygs.dsmz.de) was used to determine the closest *Bacillus* species. The analysis confirmed that strains 6E9 and 5D5 belong to *Bacillus velezensis*, while strains 2E7, 1A11, and 1H10 were identified as *Bacillus subtilis*.

## Data Availability

The draft genome sequences of *Bacillus velezensis* 6E9 and 5D5 and *Bacillus subtilis* 1H10, 2E7, and 1A11 were deposited in GenBank under BioProject PRJNA1252710. This Whole Genome Shotgun project has been deposited at DDBJ/ENA/GenBank under the accessions JBNHMZ000000000.1 (6E9), JBNHMY000000000.1 (5D5), JBNHMV000000000.1 (1H10), JBNHMX000000000.1 (2E7), and JBNHMW000000000.1 (1A11), with BioSample IDs SAMN48054946 (6E9), SAMN48054947 (5D5), SAMN48054948 (1H10), SAMN48054949 (2E7), and SAMN48054950 (1A11). The raw sequencing reads have been submitted to the SRA database and are available under accession numbers SRS24780036 (6E9), SRS24780037 (5D5), SRS24780038 (1H10). SRS24780039 (2E7), and SRS24780040 (1A11). The versions presented here represent the first releases of 6E9, 5D5, 1H10, 2E7, and 1A11 genomes.
